# 2014’s legionnaires’ disease outbreak in portugal - an intensive care, single-center. 29 patient case series

**DOI:** 10.1186/2197-425X-3-S1-A353

**Published:** 2015-10-01

**Authors:** B Oliveira, D Nora, T Carvalho, AM Araujo, L Valente, P Gomes, J Gonçalves-Pereira

**Affiliations:** Hospital de Vila Franca de Xira, ICU, Vila Franca de Xira, Portugal

## Introduction

Legionnaires' disease, a form of severe pneumonia caused by a Gram negative aerobic intracellular bacteria, was first recognised during an American Legion convention in Philadelphia in July, 1976 [1].

The second largest outbreak of Legionnaires' disease ever registered, encompassing 334 microbiologically documented cases was identified between 7 and 30 of November 2014 in Vila Franca de Xira, Portugal [2] (more than three times the usual annually reported incidence of the disease in the whole country). We report our case series of 29 critically ill patients with Legionella pneumonia admitted to the Hospital Vila Franca de Xira intensive care unit (ICU), located at the epicentre of the outbreak.

## Objectives

To report the epidemiology, clinical features, outcome, treatment strategies, complications and lessons learned during the Legionnaires' disease outbreak in an ICU setting.

## Methods

Prospective collection of clinical and epidemiological data of patients admitted with microbiologically documented Legionella pneumonia to the ICU. This data collection was started at the first day of the outbreak, after admission of 5 consecutive patients with the disease.

## Results

During the outbreak 334 patients with pneumonia were admitted through the emergency room and all either had a positive urinary antigen, culture or serology for Legionella. As much as 29 of the most severe patients were admitted to Hospital Vila Franca de Xira ICU. The main cause of admission was respiratory failure (97%), although more than half also presented renal or even multi-organic failure.

Survivors had a median ICU length of stay of 4.48 days (Interquartile range (IQR) 3-5) and a median hospital length of stay of 14.2 days (IQR 11-18.5). Overall there were four deaths in the ICU, a case fatality of 13.7%. Mean duration of hospital stay before death was 2.25 days. Patients were treated with high dose levofloxacin (1.5g/day). Non-responders also received rifampicin after 72 hours [3]. One patient experienced seizures and levofloxacin was replaced by azithromycin. A surprisingly high new incidence of atrial fibrillation (20% of patients), which was not previously described, was noted.

## Conclusions

A Legionella pneumonia outbreak was registered in November 2014 in Portugal. Several patients needed intensive care mostly for respiratory failure. A new-onset atrial fibrillation was the most common complication noted.
